# Liver Abscess Caused by *Klebsiella pneumoniae* Originating from a Peri-Implant Abscess Following Dental Implant Surgery in a Patient with Diabetes: Case Report

**DOI:** 10.3390/jcm14217634

**Published:** 2025-10-28

**Authors:** Yu-Mi Lee

**Affiliations:** 1 Department of Infectious Diseases, Kyung Hee University Hospital, Kyung Hee University College of Medicine, 23, Kyungheedae-ro, Dongdaemun-gu, Seoul 02447, Republic of Korea; cristal156@hanmail.net; Tel.: +82-2-958-8209; Fax: +82-2-968-1848; 2 Department of Infectious Diseases, Kyung Hee University School of Medicine, 23, Kyungheedae-ro, Dongdaemun-gu, Seoul 02447, Republic of Korea

**Keywords:** case report, *Klebsiella pneumoniae*, liver abscess, peri-implant abscess, pneumonia

## Abstract

Backgroud: Liver abscesses caused by *Klebsiella pneumoniae* associated with dental implant surgery are rare. We report a case of liver abscess and septic pneumonia caused by *K. pneumoniae* in a patient who developed a peri-implant abscess following dental implant surgery. Methods: The 69-year-old male patient underwent a dental implant surgery on the upper right first premolar 10 days prior to admission, and the toothache and facial swelling worsened 5 days before admission. Results: One day before admission, a peri-implant abscess was diagnosed at a local dental clinic, and the patient underwent irrigation and drainage. On the day of admission, the patient visited our dental department and was admitted through the emergency room due to melena. The patient underwent further irrigation and drainage of the peri-implant abscess around the upper right first premolar. A liver abscess in segments 2/3 (3.1 cm) and septic pneumonia were diagnosed, and *K. pneumoniae* was identified in the blood and sputum cultures. The patient received antibiotic therapy (piperacillin-tazobactam, meropenem, and ceftriaxone, sequentially) without percutaneous drainage of the liver abscess due to the patient’s refusal and recovered without complications. The patient was administered ciprofloxacin for 5 months after discharge. One month after admission, the inflammatory maker returned to within reference range. The patient improved with long-term antibiotic treatment alone without drainage of the liver abscess. To our knowledge, this is the first report of a liver abscess caused by *K. pneumoniae* originating from a peri-implant abscess after dental implant surgery. Conclusions: Clinicians should be aware of the potential occurrence of liver abscesses caused by *K. pneumoniae* if signs of systemic infection persist along with peri-implant infection after dental implant surgery.

## 1. Introduction

*Klebsiella pneumoniae* is a Gram-negative bacteria that can cause a range of mild-to-severe infections. Notably, liver abscesses caused by *K. pneumoniae* are associated with hypervirulent strains that possess specific characteristics and virulence factors [1]. The K1 and K2 serotypes of *K. pneumoniae* are related to liver abscesses and exhibit enhanced virulence due to their hypercapsular and hypermucoviscous characteristics, which are linked to the presence of genes such as *magA* and *rmpA* [1]. Among the several virulence factors of this strain, capsular polysaccharides and siderophores are essential for evading host defenses and inducing severe infections [2]. In East and Southeast Asia, including Korea and Thailand, *K. pneumoniae* accounts for 80% of pyogenic liver abscess cases [3,4,5]. In North America and Europe, liver abscesses caused by *K. pneumoniae* occur sporadically but are showing an increasing trend [6]. Hypervirulent *K. pneumoniae* can cause metastatic complications, including septic pneumonia, endophthalmitis, and meningitis in approximately 11–37% of cases [5,7,8,9,10]. The mortality rate ranged from 4 to 14%, which is lower than that of non-*K. pneumoniae* liver abscess cases [5,6,11,12]. This strain affects both healthy and immunocompromised hosts [13].

*K. pneumoniae* is a commensal organism found in the oral cavity, particularly in patients with poor dental hygiene, and is a potential causative agent of odontogenic infections [14]. Dental implant surgery is frequently performed in older adults. These procedures disrupt the protective barrier of the oral cavity, leading to peri-implant infections caused by oral commensals. With a high likelihood during the implant procedure, the implant itself or the dental instruments required for the procedure can become contaminated with *K. pneumoniae*, leading to an implant infection. Peri-implant infections following dental implant surgery can lead to bloodstream infection [15]. The anatomic proximity of oral microflora to the bloodstream can promote bacteremia and systemic dissemination of inflammatory substances [15].

Liver abscesses caused by *K. pneumoniae* that develop after dental implant surgery are extremely rare. We present a case in which a peri-implant abscess caused by *K. pneumoniae* developed after dental implant surgery, leading to a liver abscess and septic pneumonia in a patient with diabetes. This study was approved by the Institutional Review Board of Kyung Hee University Hospital, Seoul, Republic of Korea (KHUH-2025-08-042), which waived the need for written informed consent.

## 2. Case Presentation

**Figure 1:**
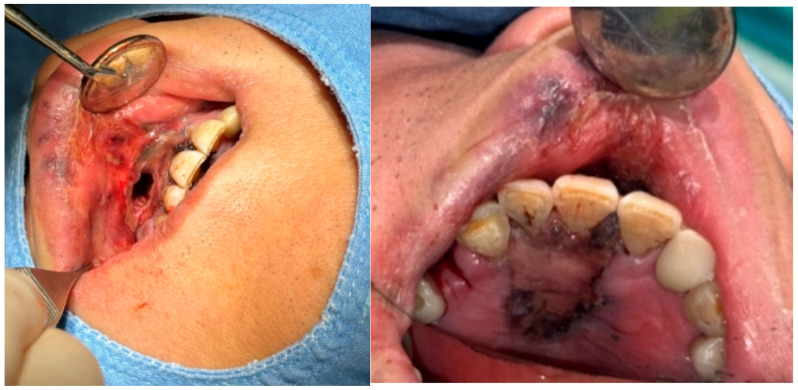
**Clinical photography of initial dental examination.** The external part of implant at upper right first premolar had already been removed at local dental clinic. Irrigation and drainage were performed at the upper right first premolar and the vestibular area of the upper right lateral incisor, canine, and first premolar. The implant fixture at the upper right first premolar was removed.

**Figure 2:**
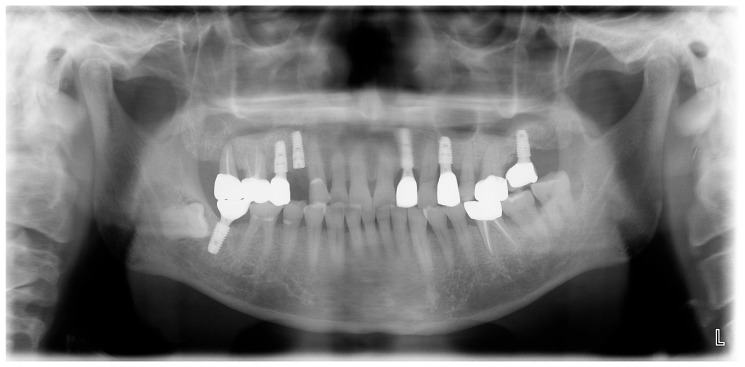
**Initial panoramic dental X-ray.** The implant fixture is placed in the alveolar bone of the upper right first premolar, and the external part has been removed.

**Figure 3:**
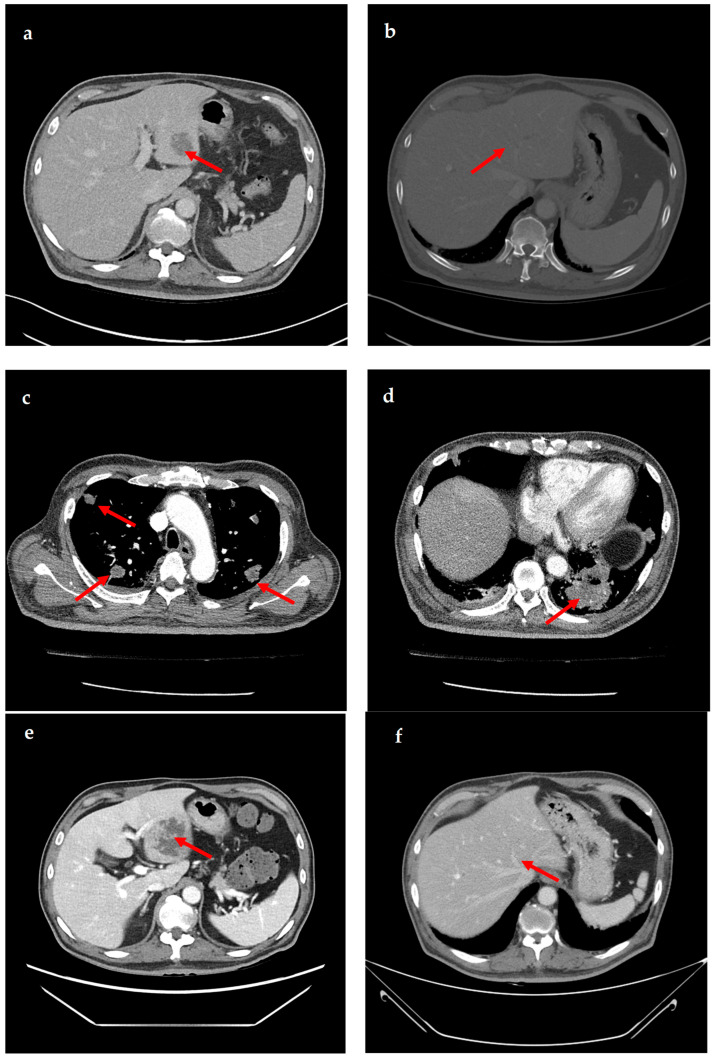
**Computed tomography (CT) images showing liver abscess and septic pneumonia.** (**a**) Gastrointestinal bleeding dynamic CT images show liver abscess (approximately 3.1 cm) at segments 2/3. (**b**) Gastrointestinal bleeding dynamic CT images show focal septic thromboembolism in the left hepatic vein. (**c**,**d**) Chest CT images show multiple mass-like consolidations in both lungs. (**e**) Abdominopelvic CT images show an increased liver abscess (approximately 6.9 cm) at segments 2/3. (**f**) Abdominopelvic CT images show that the liver abscess has resolved.

A 69-year-old male patient presenting with severe toothache 10 days before admission and melena 3 days before admission was admitted to the Infectious Disease Department of Kyung Hee University Hospital in the Republic of Korea in March 2025. The patient reported worsening toothache in the right upper gingiva with right facial swelling that began 5 days after a dental implant surgery at the upper right first premolar. Subsequently, the patient underwent incision and drainage at a local dental clinic one day before admission because of periodontitis that had spread to the facial area, as confirmed through the patient’s medical history. The patient had diabetes and was taking hypoglycemic agents. He had undergone coronary artery bypass grafting (CABG) for myocardial infarction 13 years previously. Additionally, he had undergone a prostatectomy for prostate cancer 2 years ago. He had been continuously taking clopidogrel (an antiplatelet agent) since undergoing CABG. The right buccal area of the face was swollen. Dental examination revealed a space abscess around the upper right first premolar (Figure 1). Panoramic dental X-ray showed that the implant fixture was placed in the alveolar bone of the upper right first premolar, and the external part has been removed (Figure 2). The implant fixture at the upper right first premolar was removed and irrigation and drainage were performed at the upper right first premolar and the vestibular area of the upper right second incisor, canine, and first premolar. The initial vital signs were blood pressure, 124/73 mmHg; pulse rate, 87 beats/min; respiratory rate, 20 breaths/min; temperature, 36.5 °C, oxygen saturation level, 98% on room air. Laboratory examination revealed a white blood cell (WBC) count of 6.23 × 10^9^/L (83.0% neutrophils) and a C-reactive protein (CRP) level of 417.0 nmol/L. The aspartate aminotransferase, alanine aminotransferase, and alkaline phosphatase levels were 46, 57, and 97 IU/L, respectively. The serum creatinine level was 1.08 mg/dL and the BUN was 40 mg/dL. The serum sodium level was 129 mEq/L. Plasma level of glycated hemoglobin was 13.5%. Chest computed tomography revealed a mass-like lesion in the left lung with multiple small nodules in both lungs. Two sets of blood cultures were performed on the day of admission using BD Bactec Plus Aerobic/F and BD Bactec Plus Anaerobic/F bottles and a Bactec FX Instrument (Becton Dickinson, Sparks, MD, USA). The patient underwent esophagogastroscopy, which revealed active bleeding from multiple duodenal ulcers and subsequently received hemostatic treatment. A gastrointestinal bleeding dynamic computed tomography (CT) scan showed a liver abscess at S2/3 (3.1 cm) (Figure 3a) with focal septic thromboembolism in the left hepatic vein (Figure 3b). Chest CT revealed mass-like consolidation and air densities in the left upper and lower lobes, along with multiple irregular nodules with ground-glass opacities in both lungs (Figure 3c,d).

**Figure 4:**
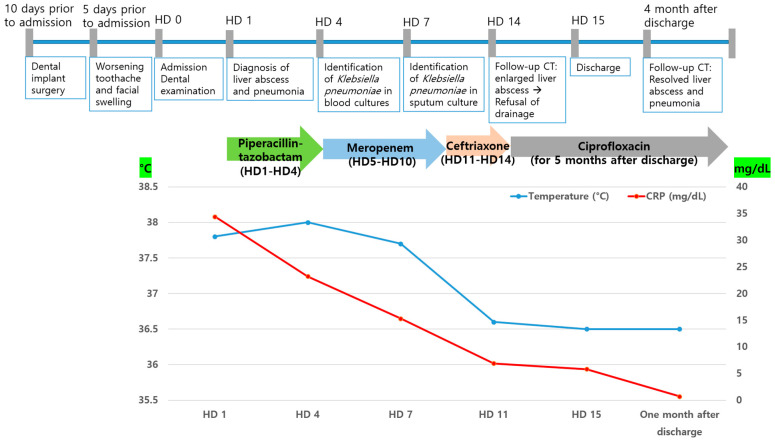
Clinical course and antibiotic treatment of a case patient. HD, hospital day, CRP, C-reactive protein.

The patient was empirically prescribed intravenous piperacillin-tazobactam (13.5 g/day). On the 4th day of hospitalization, *K. pneumoniae* was identified in two blood cultures. *K. pneumoniae* was susceptible to cefotaxime (minimal inhibitory concentration [MIC] ≤ 1 µg/mL), ceftazidime (MIC ≤ 1 µg/mL), piperacillin–tazobactam (MIC ≤ 8 µg/mL), and meropenem (MIC ≤ 1 µg/mL), but not ampicillin (MIC > 16 µg/mL). *K. pneumoniae* was also identified in the sputum culture. The patient underwent daily gingival irrigation. An ophthalmic examination revealed no signs of endophthalmitis. The patient did not have fever from the 8th day of hospitalization. It was initially switched to meropenem (3 g/day) due to the patient’s critical condition and administered for 6 days. Once the patient stabilized, the antibiotic treatment was switched to ceftriaxone (2 g/day) and administered for 4 days. On the 14th day of hospitalization, follow-up CT showed that the size of the liver abscess had significantly increased from 3 cm to 6.9 cm (Figure 3e). Drainage of the liver abscess was considered; however, the patient refused, and because the abscess was approximately 3 cm in size, only antibiotic treatment was maintained. On the 15th day of hospitalization, the patient was discharged at his request, and treatment was continued on an outpatient basis. The patient was administered ciprofloxacin (1000 mg/day) for approximately 5 months after discharge. One month after admission, the WBC count and CRP level returned to within reference ranges. Four months after discharge, follow-up CT showed that the liver abscess and septic pneumonia had resolved (Figure 3f). The clinical course and antibiotic treatment of a case patient are shown in Figure 4.

The progress of the peri-implant abscess was monitored through regular visits to the dental clinic after discharge. Alveolar bone necrosis progressed in the area of the upper right lateral incisor, canine, and first premolar due to osteomyelitis. The remaining teeth were extracted, and a sequestrectomy was performed. A bone defect remained, but healing was in progress.

## 3. Discussion

*K. pneumoniae* is a common causative pathogen of liver abscesses with metastatic complications [1]. We report a case of liver abscess with septic pneumonia caused by *K. pneumoniae* that developed alongside a peri-implant abscess in a patient who underwent dental implant surgery.

Dental implants have been covered by the National Health Insurance in South Korea since 2014. This has led to a rapid increase in the number of dental implant surgeries performed for the treatment of tooth loss. However, dental implant surgeries can lead to infectious complications [16]. Infections associated with dental implant surgery develop in 4–10% of patients receiving dental implants [17,18]. Gram-positive facultative flora primarily form colonies shortly after implant surgery [19]. However, if there is bone loss or pocket formations around the implant, colonies are formed at a high proportion by Gram-negative anaerobic bacteria, such as fusobacteria, *Prevotella intermedia*, or spirochetes [19]. Infections associated with implant surgery are predominantly caused by these colonizing bacteria [16]. Infectious complications after dental implant surgery can occur in two periods: postoperatively before prosthetic loading and after implant loading [18,20]. Infections can occur during the osseointegration procedure, prior to prosthetic loading. The presence of pus or a fistula at the surgical site indicates the occurrence of an infection. Severe peri-implant infections can lead to the loss of supportive bone around the implant [21,22]. The patient in the present case developed a peri-implant abscess after implant loading.

Prophylactic antibiotics in dental implant surgery can help reduce complications, including early implant failure and infection [23,24]. However, since dental implant surgery is often performed on relatively healthy individuals without underlying medical conditions, the routine use of prophylactic antibiotics remains controversial [25]. Previous reports showed that many dentists do not prescribe prophylactic antibiotics before dental implant surgery [25,26]. The recommended prophylactic antibiotic is a single dose of 2 g amoxicillin. However, since over 70% of *K. pneumoniae* is known to be resistant to ampicillin, the recommended prophylactic antibiotic might not prevent *K. pneumoniae* infection [27,28]. Nevertheless, the use of prophylactic antibiotics before implant procedures in high-risk groups should be emphasized. Additionally, it is important to conduct culture tests at the implant site and provide close follow-up if there are signs of infection at the implant site and accompanying systemic symptoms.

Pyogenic liver abscesses of odontogenic origin are rare. *K. pneumoniae* is a part of the oral microflora [14], and it is presumed that peri-implant infections caused by this bacterium during dental implant procedures lead to bloodstream infections. Another strong possibility is that, as an enterobacterium, *K. pneumoniae* may have contaminated the implant prior to placement, or the dental instruments and surgical drills before or during the procedure, leading to a peri-implant abscess and subsequent systemic infection. There are many reports indicating that localized odontogenic infections can spread systemically, leading to serious infections such as central nervous system infection, mediastinitis, and endocarditis [29,30,31,32,33]. There are several reports demonstrating that even minor injuries, such as tooth brushing, can lead to bloodstream infections caused by oral commensal bacteria [34]. This is due to the high density of blood vessels beneath the oral mucosa [34]. In the present case, we believe that the bloodstream infection caused by *K. pneumoniae* originating from a peri-implant abscess after dental implant surgery subsequently resulted in liver abscess and septic pneumonia. Luis et al. reported a pyogenic liver abscess caused by *Streptococcus anginosus* and *Prevotella denticola* derived from untreated dental disease [35]. A case of a liver abscess caused by *Fusobacterium necrophorum* associated with a periodontal bacterial infection has also been reported [36]. These reports emphasize that dental evaluations should be conducted when a liver abscess is caused by oral commensal bacteria. Gungor et al. documented a case of pyogenic liver abscess caused by *Streptococcus* species following the implantation of a dental prosthesis [37]. There are no reports of liver abscesses caused by *K. pneumoniae* associated with dental implant surgery. However, there have been reports of systemic infections caused by *K. pneumoniae* following odontogenic infections. Sookdee et al. documented gas gangrene in the deep neck space caused by *K. pneumoniae* in a patient with diabetes that was associated with multiple dental caries [38]. A case of splenic abscess caused by *Klebsiella ozaenae* originating from an odontogenic infection in a patient with sickle cell disease has also been reported [39].

One limitation of our study is that we were unable to perform a pus culture of the peri-implant abscess. However, given the circumstances, it was presumed that the multiple infections occurring simultaneously were caused by the same bacterium. This case highlights the fact that peri-implant infections occurring during implant procedures can lead to systemic infections, with a risk of severe infections such as liver abscess, pneumonia, meningitis, and endophthalmitis caused by *K. pneumoniae*. Early recognition that oral infections can be the source of systemic infections is important. Special attention should be given to signs of systemic infection in patients with a history of dental implant surgery. If symptoms related to systemic infection caused by *K. pneumoniae* occur, prompt testing and early treatment are crucial.

## 4. Conclusions

We present a case of liver abscess with septic pneumonia caused by *K. pneumoniae*, which was associated with a peri-implant abscess following dental implant surgery. This case suggests that liver abscesses and metastatic infections caused by *K. pneumoniae* may occur after dental implant procedures. Therefore, it is important to adhere to sterilization protocols during implant procedures and promptly notice any signs of infection after implant surgery, while paying close attention to the potential for systemic infections.

## Data Availability

The data that support the findings of this study are available on from the corresponding author upon reasonable request.

## Abbreviations

The following abbreviations are used in this manuscript:

CABG	Coronary artery bypass grafting
WBC	White blood cell
CRP	C-reactive protein
CT	Computed tomography
MIC	Minimal inhibitory concentration
